# Voltage-Dependent Anion Channel 1(VDAC1) Participates the Apoptosis of the Mitochondrial Dysfunction in Desminopathy

**DOI:** 10.1371/journal.pone.0167908

**Published:** 2016-12-12

**Authors:** Huanyin Li, Lan Zheng, Yanqing Mo, Qi Gong, Aihua Jiang, Jing Zhao

**Affiliations:** Department of Internal Neurology, Central Hospital of Minhang District, Shanghai (Minhang Hospital, Fudan University), Minhang District, Shanghai, P.R.China; Fudan University, CHINA

## Abstract

Desminopathies caused by the mutation in the gene coding for desmin are genetically protein aggregation myopathies. Mitochondrial dysfunction is one of pathological changes in the desminopathies at the earliest stage. The molecular mechanisms of mitochondria dysfunction in desminopathies remain exclusive. VDAC1 regulates mitochondrial uptake across the outer membrane and mitochondrial outer membrane permeabilization (MOMP). Relationships between desminopathies and Voltage-dependent anion channel 1 (VDAC1) remain unclear. Here we successfully constructed the desminopathy rat model, evaluated with conventional stains, containing hematoxylin and eosin (HE), Gomori Trichrome (MGT), (PAS), red oil (ORO), NADH-TR, SDH staining and immunohistochemistry. Immunofluorescence results showed that VDAC1 was accumulated in the desmin highly stained area of muscle fibers of desminopathy patients or desminopathy rat model compared to the normal ones. Meanwhile apoptosis related proteins bax and ATF2 were involved in desminopathy patients and desminopathy rat model, but not bcl-2, bcl-xl or HK2.VDAC1 and desmin are closely relevant in the tissue splices of deminopathies patients and rats with desminopathy at protein lever. Moreover, apoptotic proteins are also involved in the desminopathies, like bax, ATF2, but not bcl-2, bcl-xl or HK2. This pathological analysis presents the correlation between VDAC1 and desmin, and apoptosis related proteins are correlated in the desminopathy. Furthermore, we provide a rat model of desminopathy for the investigation of desmin related myopathy.

## Introduction

Desminopathies, also known as genetically skeletal myopathy and/or cardiomyopathy, is the most common subtype of protein aggregated myopathies. This disease ranged from childhood to late adulthood is severely disabling disease, was due to the mutation of human desmin gene on chromosome 2q35 [1]. Most of patients with desminopathy loss their kinetism for 10 to 20 years of pathogenesis and died of cardiomyopathy or respiratory failure. The muscle pathology is characterized by enlarged myofibrillar diameter, desmin protein aggregate. The precise pathology of myopathies is myofibrillar intermediate sediments. Desminopathies are mainly reported in Northern American, less in China and Japan.

Desminis an important muscle-specific type III intermediate filament cytoskeletal protein, containing the 470 amino acids. Its spherical ends mainly interact with intracellular proteins and a variety of substances and the middle rod-shaped are mainly α- helix cytoskeleton[2]. Protein filaments within the muscle cells formed skeleton, and are widely connected to the fascia, Z-disc, nucleus, mitochondria, lysosomes, endoplasmic reticulum, Golgi apparatus and other structures. Meanwhile, special spiral-helix domain of desmin molecule can play a perfect role in power transmission, so that when the muscle fiber contraction or relaxation, the junction cytoskeleton structure will constitute an integrated whole body, muscle cells and organelles make their effective adaptation in systolic and diastolic changes[3]. Mutant desmin will lose the function of the previous frame, resulting in not only mechanical power transmission dysfunction of myocyte, but also the obstacle of the mechanical signals of different molecules within the cells and myocytes. However, why the desmin gene mutations lead to abnormal accumulation of more than 60 proteins appeared in muscle cells, muscle cells, and what kinds of pathophysiological changes in organelles happened after desmin mutation, these problems have still not been fully understood clearly[4].

Mitochondrial dysfunction in the development of the disease has been the research highlights. It has been confirmed, and mitochondria are the principal target of pathological mechanisms involved in a variety of inflammatory muscle disease and hereditary muscle disease[5]. Recent studies have found that after the desmin gene mutation, mitochondrial morphology and function of muscle cells also appeared abnormal pathological changes. Desmin knockout mouse muscle cells, mitochondrial dysfunction are the earliest pathological changes, mainly increased the number of mitochondria, mitochondrial swelling and abnormal accumulation in the muscle membrane, mitochondrial membrane potential dissipation, and decreased activity of the mitochondrial respiratory chain enzyme complexes. In the mutant desmin L345P transgenic mice, the muscle fibers within the mitochondrial function and morphology also showed abnormal mitochondrial Ca^2+^levelsincreased considerably apoptosis start the mitochondria-mediated pathway at the same time[6]. Schroder R et al. found that mutant desmin K239fsX242 patient muscle cells appear abnormal distribution of mitochondria, the activity of mitochondrial respiratory chain enzyme complexes I and IV decreased[7]. Above clinical and basic studies have shown that abnormal muscle mitochondria is one of the earliest pathological changes in the desmin mutations, not only for structural mitochondrial abnormalities and abnormal position, but also, the performance of the energy metabolism and Ca^2+^dysregulation.

Mitochondrial voltage-dependent anion channel (VDAC), also called mitochondrial membrane pore protein, is an imperative mitochondrial membrane permeability material transport channel [8]. The main functions of VDAC include the following aspects: (1) VDAC controls the transport of metabolites between the mitochondria and cytoplasm, VDAC polymers form channels to modulate ATP transportation the outer mitochondrial membrane; (2) VDAC is an important part of mitochondrial permeability transformation channels (permeability transition pore, PT hole), PT hole is composed of mitochondrial proteins and cytoplasmic proteins (including: VDAC, creatine kinase, adenylate transporter, cyclophilin D, etc.), mainly localised in the inner mitochondrial membrane and the outer membrane contact sites. cytochrome C (CytoC), apoptosis inducing factor (AIF), Smac/DIABLO, nucleic acid within various apoptotic factors, endonuclease G, etc. were released into the cytosol when the mitochondrial PT pore are opening; (3) VDAC is the important protein cross-talk between mitochondria and endoplasmic reticulum, VDAC connected to endoplasmic reticulum calcium release channel IP3R physically with chaperone GRP75 (glucose-regulated protein 75) and play an important regulatory role in modulating the inner mitochondrial Ca^2+^ levels. In addition, VDAC connected to the mitochondria and endoplasmic reticulum through phosphorylation cluster sorting protein 2 (PACS2), regulating Bid of Bcl2pro-apoptotic factor family mediated apoptotic pathways; (4) VDAC is involved in the regulation of intracellular redox substances. On the one hand, VDAC can promote mitochondrial free oxygen radicals to release into the cytoplasm, on the other hand, nitric oxide (NO) and VDAC interacts directly, inhibits VDAC channel permeability, thereby inhibiting the opening of the mitochondrial PT pores [9]. In conclusion, VDAC is the important intersectional target of mitochondrial regulation of cell survival and death pathways [10].

Apoptosis is completed through the extrinsic pathway and intrinsic pathway, the extrinsic pathway is considered to be leaded by a number of extracellular signals, such as Fas or tumor necrosis factor (TNF); intrinsic pathway usually begins with the mitochondria response to different stimuli[11]. VDAC is uniquely positioned in the outer mitochondrial membrane channel, control crosstalk between mitochondria and other parts of cell metabolism. VDAC is a powerful lever in the regulation of mitochondrial metabolism [12].There are a lot of proteins interacting with VDAC1 in the cell, adjusting the permeability of PT pore and the release of the apoptotic material of mitochondrial membrane gap, thereby regulating cell apoptosis. The bcl-2 protein family is one of the key factors in the regulation of apoptosis, and plays an important role in apoptosis signal transduction pathway. Bcl-2 and bax are the most representative the anti-apoptotic and pro-apoptotic genes of bcl-2 family respectively, and bax is the main regulator of bcl-2 activity [12]. bax can interact with VDAC to increase VDAC aperture and increases mitochondrial permeability, promoting apoptosis [13]. The bcl-2 protein is an essential anti-apoptotic proteins, preventing the release of cytochrome C and the activity of caspase. The proper proportion of bax and bcl-2 maintains the cell homeostasis to ensure cell survival [14].

In the studies of tumor cells, it was found that tumor cells still maintain energy supply even in the presence of oxygen by enhancing the glucose anaerobic glycolysis, this enhanced characteristics of tumor cell is called aerobic glycolysis tile Berg effects (Warburg effect) [15]. Further studies showed that the conjunction of mitochondrial and hexokinase is the key of Warburg effect [16,17]. Additional studies showed that hexokinase (HK) and mitochondrial outer membrane through VDAC associated [18]. There are four genes encoding four kinds of HK subtypes in the human genome. HKⅠand HKⅡare positioned in the outer mitochondrial membrane.HKⅢis localized in the nucleoside, HKⅣis localized in the cytoplasm [19]. Evidences suggest that HKⅡof four subtypes is really related to VDAC in 1998 [20]. HKⅡand VDAC mitochondrial membrane gap prevents the release of binding protein and apoptosis [12].

Evidence suggested that activated into factor (ATF2) of cytoplasmic localization is associated with the cell death and stress of disease process [21]. Genotoxic stress stimulates nuclear ATF2 to translocate to the cytoplasm. ATF2 within the cytoplasm is positioned on the outer membrane of mitochondrial, and then mitochondrial membrane potentially reduced, causing mitochondrial depolarization, which increases the permeability of mitochondria, causing mitochondrial-dependent cell apoptosis. ATF2-Bim-VDAC1 hierarchically regulates apoptosis, ATF2 damages HK-VDAC1 complex, inspires Bim-mediated BAX-VDAC complex formation to increase the permeability and the release of mitochondrial cytochrome C, resulting in induction of apoptosis [22].

In mammals, VDAC have three kinds of isoforms: VDAC1, VDAC2 and VDAC3, which VDAC1 is the most abundant and mainly studied. In HeLa cells, the amount of VDAC1 is ten times more than VDAC2, 100 times more than VDAC3 [23].

Desmin is the most important protein in muscle cells intermediate filament cytoskeletal.Whether there is interaction between desmin and VDAC, and when desmin changes, what changes of VDAC function will occur, how mitochondrial happen to change, it is not clear. The study adenovirus coated protein gene mutation junction constructs desmin disease animal model, by contrasting immunohistochemical results of desminopathy patient-related diseases and animal models of desminopathy disease, we found that desmin mutation and VDAC1, bax, bcl-2, HKⅡ, ATF2 are closely related, it provided ideas for the study of other neurological protein aggregation diseases, also provides a new approach for drug development.

## Materials and Methods

### Cell and Regents

HEK-293 cells were purchased from ATCC (USA).Incision enzymes were purchased from NEB Company and Takara Company. LR ClonaseⅡwas bought from Invitrogen. METAFECTENE^TM^ (Biotex, USA) were used for transduction in HEK-293 cells.

### Construction of rAd5-DES (pad-DES)

Adenovirus vectors ofrAd5-DES were performed by Jingsai biotechnology (Wuhan, China), coated with mutation protein of *DES* at c.821T>C; L274P (S5 Fig). Vectors of pad-DES were transferable intoHEK-293 cells with METAFECTENE^TM^ followed the manufactural instruction. The titer of adenovirus was obtained by Tissue Culture Infectious dose 50 Assay.

### Ethics statement

All animal works were conducted according to relevant national and international guidelines. They were approved by the Committee on the Ethics of Animal Experiments of Shanghai Jiao tong University and monitored by the Department of Experimental Animals of Shanghai Jiao tong University. Clinical specimens of desminopathy patients were acquired by surgery or biopsy from desminopathy patients, which were approved by the Ethical Committee of Central Hospital of Minhang District of Shanghai. Written Informed consent was provided by all patients in this study.

### Animal experiment

Male SD rats (18–25 days old) were supplied by the Animal Scientific Department of Shanghai Jiaotong University. The rats were maintained in the accredited animal facility of Shanghai Jiao tong University, and animal care was in accordance with institutional guidelines. The rats were received intramuscular injection with rAd5-DES (10^10^pfu per rat) at the left legs with 9 palace grids method, every grid is 0.5cm×0.5cm (S6A Fig),and accordingly the rats were received intramuscular injection with the same voltage of normal saline as control. The rats run in a wheel at the time of 6 and 9 o’clock everyday (S6B Fig). Rats were sacrificed at d14 after injection. The muscle tissues of left or right legs were collected and stored in the -80°C. The skeletal muscle was cut by using CM1950 frozen section machine (Leica, Germany) and prepared for the Conventional stain or Immunohistochemistry.

We have any efforts to alleviate suffering for rats used in this study as the following steps: Anesthesia: The chloral hydrate was formulated into 5% solution with sterile saline. Rats were received intraperitoneal injection of 5% chloral hydrate following 0.6ml / 100g, and the anesthetic into three equal parts, injection of 1each at interval 3min, observe the effect before the third injection, cancel the third injection if it has entered the anesthesia; and the injection continues if it fails to reach anesthesia. After injection of narcotic drugs, pull out the needles lightly to avoid leakage. Two rats of each were sacrificed respectively at the time points: 2 days, 7 days 14 days, 21 days or 28 days. After we accessed the rat model of different time points, 14 days were chosen in this model for further immunohistochemistry and immunofluorescence analysis. Eight rats of each group were performed in the further experiment. The rats were received intramuscular injection with rAd5-DES (10^10^pfu per rat) at the left legs with 9 palace grids method.

#### Participant recruitment and patient tissue collection

Select the patients(S1 Table) diagnosed with desmin myopathy in the neurological department of our hospital between October 2014 and January 2016, and the patients with the same electrophysiological neuromuscular symptoms and muscle biopsy but the normal final diagnosis in the same period, were set as the control group. After obtaining the approval of the hospital ethics committee, and patients and their families signed the informed consent of patients and routine physical examination, an open surgical incision was selected at the left biceps biopsy site after local anesthesia to obtain muscle specimens between October 2014 and January 2016.

### Histology

#### Conventional Stain

The frozen sections of the skeletal muscle of desminopathy patients and desminopathy rat model were prepared for conventional stain, containing hematoxylin and eosin (HE), Gomori Trichrome (MGT), (PAS), red oil (ORO), NADH-TR, SDH stain, the method of these staining followed the previously published [24].

#### Immunohistochemistry and immunofluorescence

Tissue sections were prepared and subjected to immunohistochemical analysis or immunofluorescence. Murine anti-desmin Ab (Abcam) and Goat anti-murine second Ab (DAKO) were used for immunohistochemistry. Murine anti-desmin monoclonal Ab (Abcam), Rabbit anti-bcl-xl monoclonal Ab (Abcam), Rabbit anti-ATF2 polyclonal Ab (Abcam), Rabbit anti-bax polyclonal Ab (Protein Tech), Rabbit anti-HK2 polyclonal Ab (Protein Tech), Rabbit anti-bcl-2 polyclonal Ab (GeneTex), TRITC-labeled goat anti murine IgG (Protein Tech) and FITC-labeled goat anti rabbit IgG (Protein Tech) were used for immunofluorescence. Images were obtained using a NIKON-Ds-RIL microscope at 40 x10 magnifications. Statistics of these proteins were analyzed by using Image-pro plus 6.0.

## Results

### Pathological characteristics of patients with desmin-related myopathy

**Fig 1:**
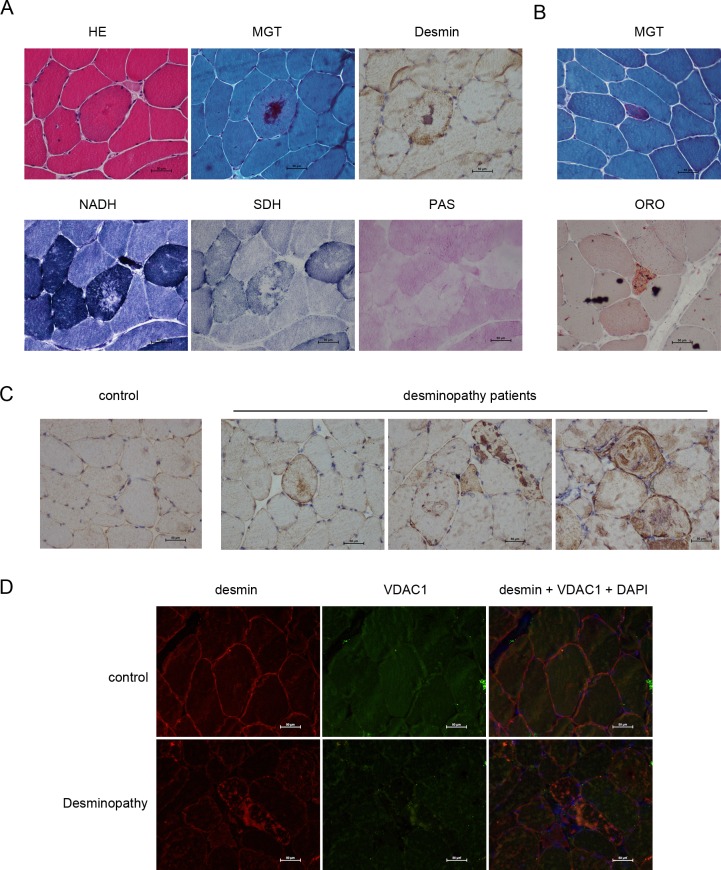
VDAC1 is involved in the desminopathy patients. The skeletal muscle fibers of desminopathy patients were treated with HE, MGT, immunohistochemical, NADH, SDH or PAS stain **(A)**; individual atypical RRF and individual lipid droplets by MGT and ORO stains were shown in **(B)**; desmin in the muscle fibers of patients were detected by immunohistochemistry as shown in **(C)**, the muscle fiber of normal person was set as a control **(C)**; VDAC1 and desmin in the muscle fibers of desminopathy patients were analyzed by immunofluorescence as shown in **(D)**. Scale bars, 50μm.

Firstly, we collected the frozen sections of the left biceps of patients with desmin-related myopathy, then analyze by using routine staining and immunohistochemical staining. It suggested that the border of muscle cross-section is still clearly displayed and mild hyperplasiain the connective tissues within the muscle bundle. Small blood vessels were normal without significant cardiovascular infiltration of the inflammatory cells and deposits of abnormal materials. There are some angular muscle fibers within the bundle loosely arranged due to the proliferation of internal connective tissues.The muscle fiber diameters displayed the unimodal distribution. Normal muscle fiber diameter is between 30–90 microns, and the group scattered distribution of small circular or angular muscle fiber atrophyhas a diameter of between 5–30 microns. Little muscle fibers hypertrophy occurs; the maximum diameter is 110 microns. Eosinophilic materials appeared in the visible part of the muscle fibers lumps or patchy distribution, the phenomenon of some muscle fibers shift in the nucleus occurs (Fig 1A), there are rimmed vacuoles within few muscle fibers. Individual muscle fiber necrosis, necrotic muscle fibers with the old phagocytic cell infiltration, and necroticfresh-like muscle fibers showed homogeneous changes. Basophilic material deposited appears in broken individual muscle fiber therein. We found no muscle membrane-like changed fibers without regeneration occurred, or cyclic, spiral and split changed. Homogeneous substances and visible individual atypical RRF (Fig 1B) occurred within few muscle fibers. The fat droplets increased substantially inindividual muscle fibers (Fig 1B). PAS staining showed no stained muscle fibers and lack of glycogen substance in abnormal material deposition zone (Fig 1A). NADH-TR staining showed few stained atrophic muscle fibers, the material deposited activity within abnormal muscle fibers appeared flaky deletion (Fig 1A). SDH staining showed that the lack of activity in the central portion of the muscle fibers, and a small amount of residual activity (Fig 1A) at its periphery. NSE staining showed the lesions visible stained part of muscle fibers and infiltration of inflammatory cells. Immunohistochemical staining of desmin revealed that corresponding positive deposits also emerged in the majority of MGT stained muscle fibers (Fig 1A).

Normal desmin displayed grid-like pulp diffuse expression under sarcolemma and package (Fig 1C and S1A Fig), while desmin aggregated in muscle fiber of patients with desminopathy, which appeared the following characteristics: (1) diffuse positive aggregation within the package pulp or under sarcolemma (Fig 1C and S1A Fig); (2) strong positive lumpy deposits in the package pulp, pellet-like deposition (Fig 1C and S1A Fig); (3) strongly positive irregularly aggregates in the pulp (Fig 1C and S1A Fig). VDAC1 is involved in desmin aggregation in the patients with desminopathy.

Immunofluorescence analysis of desminand VDAC1 in the frozen sections of left biceps of patients with double detection of muscle fibers within desmin strong positive irregular mass gathering like high signal (Fig 1D and S1B Fig), VDAC1 fluorescent display appears VDAC1 high in the corresponding muscle fibers signal aggregates (Fig 1D and S1B Fig); Skeletal desmin in muscle membrane, evenly distributed in the cytoplasm (Fig 1D and S1B Fig), VDAC1 normally evenly distributed in the cytoplasm (Fig 1D and S1B Fig).

### Apoptosis related proteins are involved in the desmin aggregation of the patients with desminopathy

**Fig 2:**
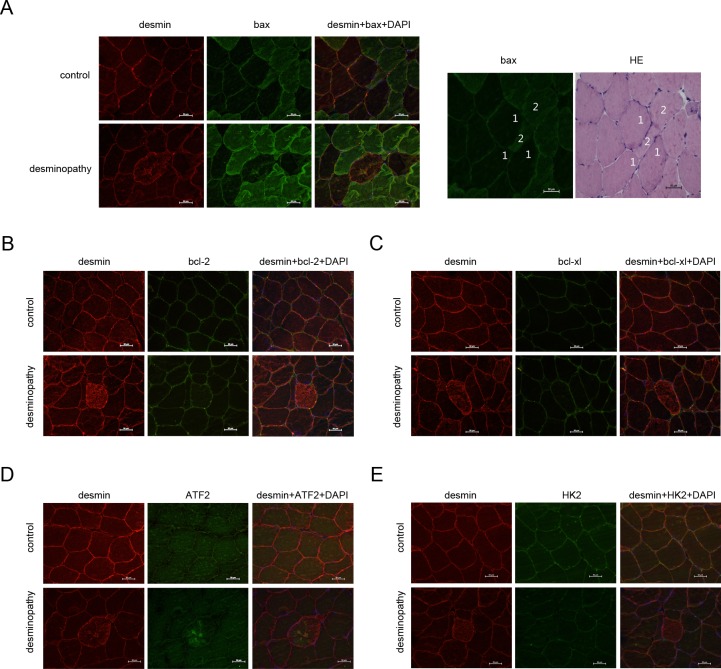
Apoptosis related proteins are involved in the desminopathy patients. The apoptosis related proteins bax **(A)**, bcl-2 **(B)**, bcl-xl **(C)**, ATF2 **(D)** and HK2 **(E)** of normal person **(control)** or desminopathy patients were detected by immunofluorescence; in the normal muscle fibers, bax was lightly stained in type 1 muscle fiber, and deeply stained in type 2 muscle fiber as shown in **(A, right).** Scale bars, 50μm.

VDAC1 are closely related to the apoptosis of cells, especially bax interacts with VDAC1 to enhance the permeability of mitochondria, and then promote the apoptosis. Bcl-2 is an anti-apoptotic protein, which maintains the survival of cells with bax at the proper percentage. ATF2 forms a complex ATF2-Bim-VDAC1, which regulates the apoptosis. So we analyze bax, bcl2,bcl-xl, and HK2 expression and location in the muscle fibersof patients with desminopathy by using immunofluorescences. Fluorescent desmin displayed irregular aggregation high signal (Fig 2 and S2 Fig) muscle fibers, bax fluorescent display high signal accumulation also appear in the corresponding muscle fibers (Fig 2A and S2 Fig); Skeletal desmin in muscle membrane, evenly distributed in the cytoplasm (Fig 2A and S2 Fig); bax evenly distributed within the normal muscle fibers, muscle fibers showed a mosaic-like distribution (Fig 2A and S2 Fig). Immunofluorescence staining of bax in muscle fibers appeared the mosaic distribution of lightly stained, stained (Fig 2A and S2 Fig), corresponding to HE staining (Fig 2A, right and S2 Fig) type 1, type 2 muscle fibers. bax fluorescence was lightly stained in Type 1 muscle fibers and thickly stained in type 2 muscle fibers.

Fluorescent desmin showed diffuse hyperintensity in the muscle fibers of patients(Fig 2B and S2 Fig), bcl-2 fluorescence display no significant abnormality between fluorescence the corresponding muscle fibers and normal muscle fibers(Fig 2B and S2 Fig); desminevenly distributed in muscle membrane and the cytoplasm of normal skeletal muscle (Fig 2B and S2 Fig), bcl-2displayeduniformly low signal within the normal muscle fibers(Fig 2B and S2 Fig). Fluorescent desmin in muscle fibers diffusely display high signal (Fig 2C and S2 Fig), fluorescent bcl-xl display no significant differences between corresponding muscle fibers and normal muscle fibers (Fig 2C and S2 Fig); desmin evenly distributed in the normal cytoplasmand the normal muscle membrane (Fig 2C and S2 Fig), bcl-xl displayed uniformly low signal within normal skeletal muscle (Fig 2C and S2 Fig). High fluorescent desmin signal appeared in sarcolemma and their regular lumps aggregation of muscle fibers (Fig 2D and S2 Fig), high signal of fluorescent ATF2 display in the correspondingly irregular aggregation of the muscle fibers (Fig 2D and S2 Fig); Skeletal desmin in muscle membrane, evenly distributed in the cytoplasm (Fig 2D and S2 Fig), ATF2 distributed evenly in the cytoplasm of normal human skeletal muscle (Fig 2D and S2 Fig). Fluorescent desmin display muscle fibers diffuse hyperintensity (Fig 2E and S2 Fig), HK2 fluorescent display corresponds to the fluorescence signal than normal muscle fibers slightly reduced (Fig 2E and S2 Fig); normal desmin in muscle membrane, evenly distributed in the cytoplasm (Fig 2E and S2 Fig), HK2 within normal muscle fibers evenly distributed (Fig 2E and S2 Fig).

### Construction of the desminopathy rat model

**Fig 3:**
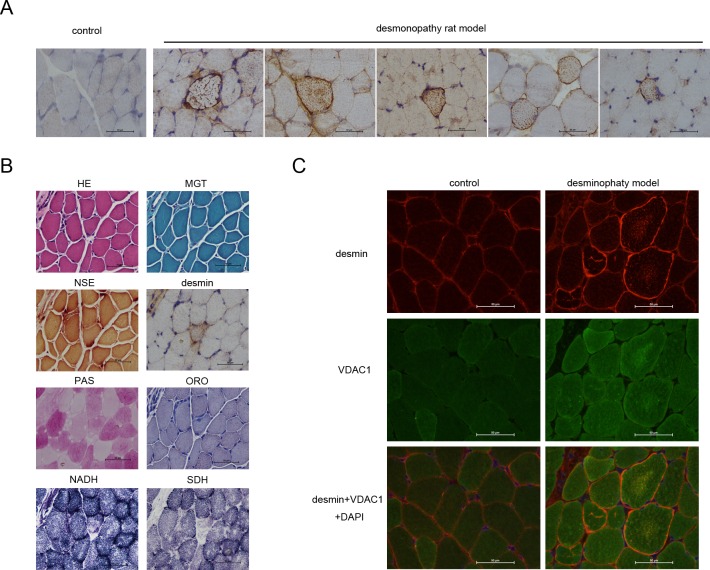
VDAC1 is involved in the desminopathy rat model. In the desminopathy rat model, normal (control) or mutant desmin(rAd5-DES) transferred muscle fibers were analyzed by immunohistochemistry **(A)**; desminopathy muscle fibers were detected by HE, MGT, NSE, immunohistochemical, PAS, ORO, NADH or SDH stain **(B);** VDAC1 and desmin in the normal or desminopathy muscle fibers were detected by immunofluorescence as shown in **(C)**. Scale bars, 50μm.

**Fig 4:**
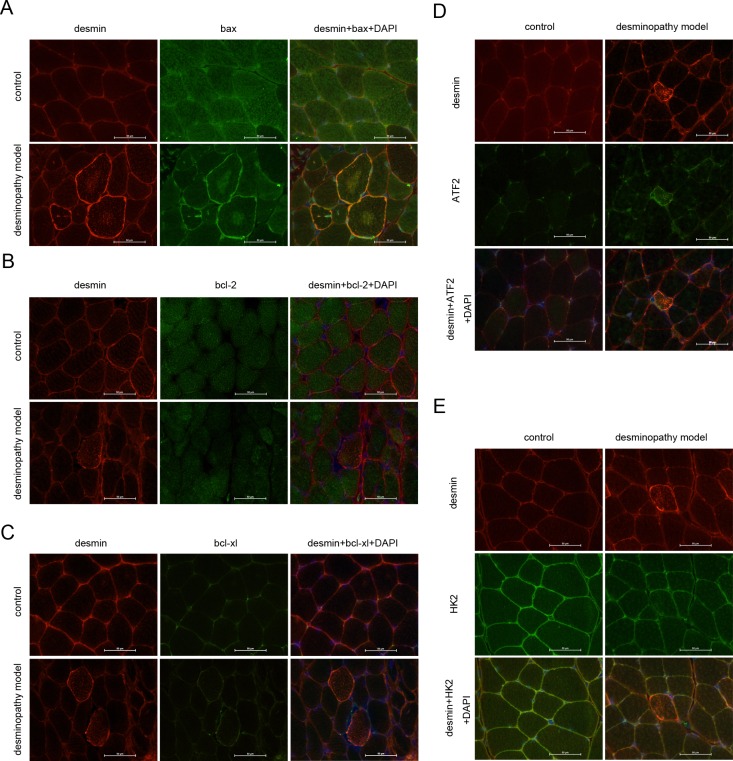
Apoptosis related proteins are involved in the deminopathy rat model. Apoptosis related proteins bax **(A)**, bcl-2 **(B)**, bcl-xl **(C)**, ATF2 **(D)** or HK2 **(E)** was analyzed by immunofluorescence in the normal **(control)** or desminopathy muscle fibers. Scale bars, 50μm.

We constructed the plasmid pad-DES which including the point mutation of *desmin*. Then pad-DES was injected into skeletal muscle of SD rats (see methods in detail). 14 days later rats were sacrificed and skeletal muscle was collected for frozen section. The desmin aggregation displayed typically in the desminopathy-like muscle fibers by immunohistochemistry (Fig 3A and S3A Fig). Then we repeated that 4 SD rats were injected with pad-DES and rats displayed the desmin aggregation in the muscle fibers (Fig 3A and S3A Fig). In this model, desmin aggregates in the muscle fibers (Fig 3B) within a small amount of occasional eosinophilic material deposition (Fig 3B). Parts of the muscle fibers shift to the nucleus with the occasional nuclear aggregation. MGT staining showed no abnormalities (Fig 4B). PAS staining showed no abnormal desmin deposition within the muscle fibers (Fig 3B). NADH staining showed the individual stained muscle fibers were atrophy; desmin deposition in muscle fibers appears the patchy lack of enzyme activity (Fig 3B). SDH staining confirmed the lack of activity in the muscle fibers (Fig 3B).

#### Correlation of VDAC1 and desmin was validated in the desminopathy rat model

Desminand VDAC1 were detected in the frozen sections of skeletal muscle specimens of model using the immunofluorescence staining, The diffuse high signal or strong positive expression of abnormal desmin regularly aggregated in muscle fibers (Fig 3C and S3B Fig), VDAC1 aggregates within the corresponding muscle fibers (Fig 3C and S3B Fig), and the normal desmin distributed in the muscle membrane or cytoplasmic (Fig 3C and S3B Fig), normal VDAC1 expressed diffuse in the cytoplasm (Fig 3C and S3B Fig).

#### Correlation of apoptotic proteins and desmin were validated in the desminopathy rat model

Desmin, bax, bcl-2, bcl-xl, ATF2 and HK2were detected in the frozen sections of muscle fibers by using immunofluorescence staining. In the abnormal muscle fibers, it showed the diffuse high signal or strong positive expression of desmin aggregationin the muscle fibers (Fig 4 and S4 Fig); sarcolemma, bax positively aggregated in pulp (Fig 4A and S4 Fig); Bcl-2 expressed weaker compare to the normal muscle fibers(Fig 4B and S4 Fig); There was no significant difference of bax expression compare to the normal muscle fibers (Fig 4A and S4 Fig); The signaling of ATF2 aggregated highly in the corresponding muscle fibers of abnormal desmin (Fig 4D and S4 Fig). There was no difference of the expression of HK2 between the normal muscle fibers and abnormal ones (Fig 4E and S4 Fig). Correspondingly, in the normal muscle fibers, desmin distributed in normal muscle membrane, evenly distributed in the cytoplasm (Fig 4 and S4 Fig); bax distributed evenly within the muscle fibers (Fig 4A and S4 Fig), where type 1 muscle fibers were lightly stained, type 2 muscle fibers were thickly stained. Bax exhibits the pale stain mosaic-like stained in the muscle fibers. Bcl-2 distributed evenly in the skeletal muscle(Fig 4B and S4 Fig the distribution of Bcl-xl was even in the muscle fibers (Fig 4C and S4 Fig). ATF2 was distributed evenly in the nucleus or cytoplasm (Fig 4D and S4 Fig). HK2 be distributed evenly in the normal skeletal muscle membrane and cytoplasm (Fig 4E and S4 Fig).

## Discussion

Desminopathyis hereditary myopathy caused by mutations of desminin the skeletal or cardiac muscle [25, 26]. In this study, we have successfully constructed desminopathy disease animal model by direct intramuscular transduction with adenovirus coding mutations of the gene *desmin*, the high stability of the model was validated. In frozen sections of skeletal muscle of the desminopathy patients or animal model, we found that desmin and VDAC1 deposition with high signals in muscle fibers, and the related pro-apoptotic proteins (bax, ATF2) also appeared high signal aggregation, and about related anti-apoptotic proteins (bcl-2, bcl-xl, HK2), HK2 in patients and bcl-2 in desminopathy rat model are slightly lower expressed than the normal ones, the other anti-apoptotic proteins are unclear. Thus, we propose the following model: (1)gathered desmin caused bax from polymerization channel in the mitochondrial outer membrane, bax and VDAC1 are combined. (2) bcl-2 was reduced caused by desmin aggregation, the ratio of bax and bcl-2 is more than 1.(3) desmin aggregation caused the increase of ATF2, stimulating the formation of bim-mediated bax-VDAC1 complex, or ATF2 was directly targeted to the outer mitochondrial membrane, reducing mitochondrial membrane potential. (4) Desmin aggregation inhibited the expression of HK2, reducing the formation of HK2-VDAC complex and increasing bax/bak formed polymerization hole channel in the outer mitochondrial membranes. High expression of VDAC1 increased the permeability of the mitochondrial membrane, then the pro-apoptotic factors released from the mitochondrial membrane gap to the cytoplasm, eventually leading to apoptosis.

In the previous study, Pierre Joanne constructed desminopathy model by direct intramuscular transduction with the protein of the mutant desmin coated adeno-associated virus [27]. Because adenoviral vector can also be transfected into the dividing or non-dividing cells, the virus titer is high and is easily prepared and purificated, does not integrate into the host cell genome, the current adenovirus has become recognized the most ideal gene vectors. In this study, desminopathy model was constructed by direct intramuscular transduction with the adenoviral vector containing mutations of desmin(c.821T> C; L274P). We successfully constructed the desminopathy rat model with intramuscular transduction and re-verify the high stability of the model. Mitochondrial dysfunction in the progress of the disease has been a highlighted topic. Muscle mitochondrial morphology and function showed the abnormal significantly increased Ca^2+^ levels in mitochondria starts mitochondria-mediated apoptotic pathway [6]. Varying amounts of cytochrome C negative (COX-) muscle fibers and not the typical ragged red muscle fibers (RRF) appeared in the muscle biopsy specimens of desminopathy patients [28]. The clinical and basic studies have shown that mutations mitochondria abnormality is one of the earliest pathological changes in the abnormal muscle with desmin mutation, not only for structural mitochondrial abnormalities and abnormal position, but also the dysregulation of the energy metabolism and Ca^2+^.

Permeability transition (PT) pore were formed by the combination of mitochondrial proteins and cytoplasmic protein comprising: a voltage-dependent anion channel (VDAC), creatine kinase, adenylate transporter, cyclophilin D etc. PT hole was mainly located in the contact sites of the mitochondrial inner membrane and the outer membrane. Cytochrome C (CytoC) apoptosis inducing factor (apoptosis inducing factor, AIF), Smac / DIABLO, a variety of apoptotic factors endonuclease G, etc. were released into the cytosol when the mitochondrial PT pore is opening and induce apoptosis; VDAC also called mitochondrial porin is an important part of PT holes, located in the outer mitochondrial membrane. Further Ca^2+^, ATP, glutamate, and many other proteins of NADH and VDAC interaction regulate the permeability of the VDAC channel[8,29]. Eukaryotic VDAC have VDAC1, VDAC2, VDAC3 subtype III, respectively, by three distinct genes encoding [8], which VDAC1 is the most studied subtype currently. In this study, patients and desmin Construction disease model, row frozen section of skeletal muscle desmin and VDAC1 double staining detected desmin accumulation in muscle fibers VDAC1 there gathered high signal. Tip desmin and muscle disease VDAC1 highly expressed genes are closely related.

There are many proteins combining with VDAC to regulate the permeability of the mitochondrial membrane and apoptosis. Bcl-2 family proteins play a key role in the regulation of apoptosis, so far, there are at least 19 kinds of proteins of human bcl-2 family are found. This family includes seven kinds of anti-apoptotic proteins:bcl-2, bcl-Xl, Mcl-1, Bcl-B, Bcl-w, A1 / Bfl1; and other pro-apoptotic proteins: Bax, Bak, Bok, bim, Bmf, Puma, Noxa, Bad, Bid, Bcl-Xs, BiNP3, Hrk, respectively [30]. Bcl-2 and bax of Bcl-2 family proteins in apoptosis are most important [31, 32]. Bax mainly distributed in the cytoplasm of the package and is shifted to mitochondrial outer membrane to form dimers, trimers, and adenylate transporter combination response to the stimulation of apoptosis signaling, or combines with VDAC directly to induce VDAC open and increases the permeability of mitochondrial membrane, thereby causing induction of apoptosis [33]. The major anti-apoptotic protein Bcl-2 is an integral membrane protein of bcl-2 family proteins, mainly anchored in the outer membrane of mitochondria, endoplasmic reticulum, cytoplasmic side of the nuclear membrane, and competitive inhibition of bax, thereby inhibiting apoptosis [34, 35]. The ratio of bax/bcl-2 regulates the apoptosis or inhibition of apoptosis, while bax / bcl-2 is more than 1, the cells tend to apoptosis; when bax / bcl-2 is less than 1, the cell s tend to anti-apoptosis [36].

Bcl-xl and bcl-2 are highly homological[37].The overexpression of bcl-xl will protect the hippocampus and cortex nerve cells against ischemia and hypoxia [38]. Under hypoxic-ischemic brain damage condition, bcl-xl gene may also inhibit caspase-3, caspase-9 activity, inhibiting the shift of apoptosis inducing factor (AIF) to the nucleus [39]. Overexpression of bcl-xl can also protect primary neurons antioxidant sugar deprivation and hypoglycemia stress in rats [40]. Studies have shown that bcl-xl plays an important role in the number of mitochondria and its division or integration [41], and also maintains the stability of mitochondrial membrane potential [42] and ATP synthase effect. Recent studies have shown that theBH4 area of bcl-xl, targeting the VDAC1, reduces mitochondrial Ca^2+^ influx mediated by VDAC1 to inhibit apoptosis [43]. Abnormal accumulation of bax appears in skeletal muscle fibers of desminopathy patients and desminopathy the content of baxin type 1 muscle fibers is lower than type 2 muscle fibers; there is no difference of bcl-2 and bcl-xl in muscle fibers withdesmin aggregation of between patients and normal people,. Within desminopathy animal models, bcl-2 signal is slightly lower in muscle fibers with desmin aggregation of animal model than normal, while bcl-xl no significant difference compared with normal. It suggested that desmin deposition in muscle fibers tends to apoptosis.

Under normal circumstances, activated into factor 2 (ATF2) can be found in the nucleus and cytoplasm. Overexpression of Cytoplasmic ATF2 predicts low malignancy and good prognosis [44]. ATF2 aggregation can be observed in the cytoplasm of prostate cancer rafter radiotherapy[45]. Overexpression of cytoplasmic ATF2 induced cell death in melanoma, thereby reducing the transcriptional activity of endogenous ATF2 [46]. Evidence suggested BH3 protein (BH3s), such as Bim, can induce BAX and VDAC restructure in the mitochondrial outer membrane to form the new channels [47, 48]. Recent studies have found that ATF2, Bim, VDAC1 hierarchically regulate apoptosis, ATF2 damages HK-VDAC1 complex, stimulating Bim-mediated the formation BAX-VDAC complex to increase permeability and the release mitochondrial cytochrome C, and induce death [22]. We have found that ATF2 deposited in the skeletal muscle fibers with desmin deposition of desminopathy patients and animal models, which suggests that desmin deposition in muscle fibers and tends to apoptosis.

HKⅡ-VDAC complexes interfere the conjunction of bcl-xl with VDAC, leading to an increase of free bcl-xl. Increased bcl-xl and bax binding inhibits the formation of bax/bak in the outer mitochondrial membrane, inhibiting apoptosis [43]. Recent studies showed that mechanistic target of rapamycin kinase inhibitor (mTOR-KI) promotes HKⅡto bind to VDAC binding to shut down the PT hole, while the dissociation of HK2 from the VDAC loss the function of mTOR-KI[49]. Our study found that HKⅡslightly weakened within muscle fibers of the desminopathy patients than normal, Indicating that the occurrence of desminopathy is related to the reduction of HKⅡ.

In this study, there are also some shortcomings. First of all, a small sample volume and the lack of large sample statistics. Secondly, it is a short time observed in this animal model, discrepancies of proteins in animal models and patients with desminopathy. Thirdly, proteins are lack of quantitative comparison in this study observed morphological changes of muscle fibers. However, we have successfully constructed desmin disease animal models with the validation of high stability of the model. Furtherly, we present the understanding of desmin related myopathies by studying the mitochondrial apoptotic proteins. All above, we presented powerful evidences that VDAC1 are correlated with the desminopathies. Moreover, apoptosis related protein are also associated with desmin mutation in the patients with desminopathies or rat desminopathies model. The deeply mechanism of VDAC1 in the desminopathies need to be furtherly explored, so as to the mitochondrial dysfunction.

## Supporting Information

S1 Fig VDAC1 participates in the desminopathy patients. **(A)**Statistical analysis of Fig 1C. **(B)**Statistical analysis of Fig 1D.
(TIF)

S2 Fig Apoptosis related proteins are correlated with desmin in desminopathy patients. Statistical analysis of Fig 2.
(TIF)

S3 Fig VDAC1 participates in the desminopathy rat model. **(A)**Statistical analysis of Fig 3A. **(B)**Statistical analysis of Fig 3C.
(TIF)

S4 Fig Apoptosis related proteins are correlated with desmin in desminopathy rat model. Statistical analysis of Fig 4.
(TIF)

S5 Fig The construction of rAd5-DES. The point mutation of desmin as shown at the *arrow* (A),mutation position: c.821T>C, L2Ad5-DES as shown in (B), M1: Wide Range 2000 Marker(Takara):2kb,1kb,750bp,500bp,250bp,100bp; M2: Wide Range 500–15000 Marker(Takara): 15kb, 8kb, 5kb, 2.5kb, 1kb, 0.5kb; p1: the identification of rAd5-DES with restrictive endonuclease XbaI; p2: repeated p1.
(TIF)

S6 Fig Desminopathyrats model. The 9 grids’ area of intramuscular injection was shown at **(A)**, then the rats run at the designed time in the wheel everyday **(B)**.
(TIF)

S1 Table The information of desminopathy patients in our hospital. (DOCX)
