# Supplementary figures and images for: Voltage-Dependent Anion Channel 1(VDAC1) Participates the Apoptosis of the Mitochondrial Dysfunction in Desminopathy
Source: PLoS One. 2016 Dec 12;11(12):e0167908. doi: 10.1371/journal.pone.0167908 (PMC5152834; doi:10.1371/journal.pone.0167908)

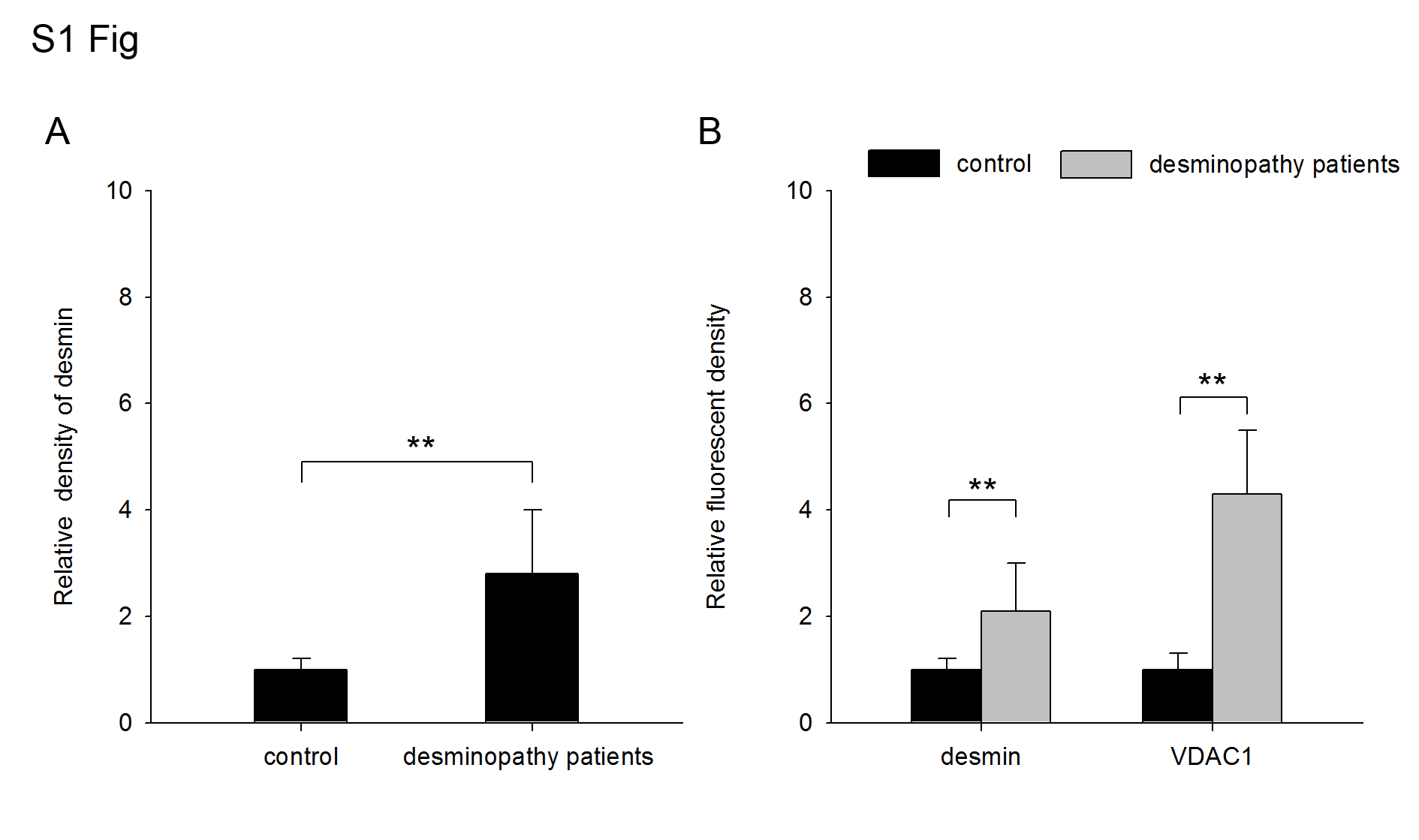

Supplement: S1 Fig — (A)Statistical analysis of Fig 1C. (B)Statistical analysis of Fig 1D. (TIF) [file pone.0167908.s001.tif]

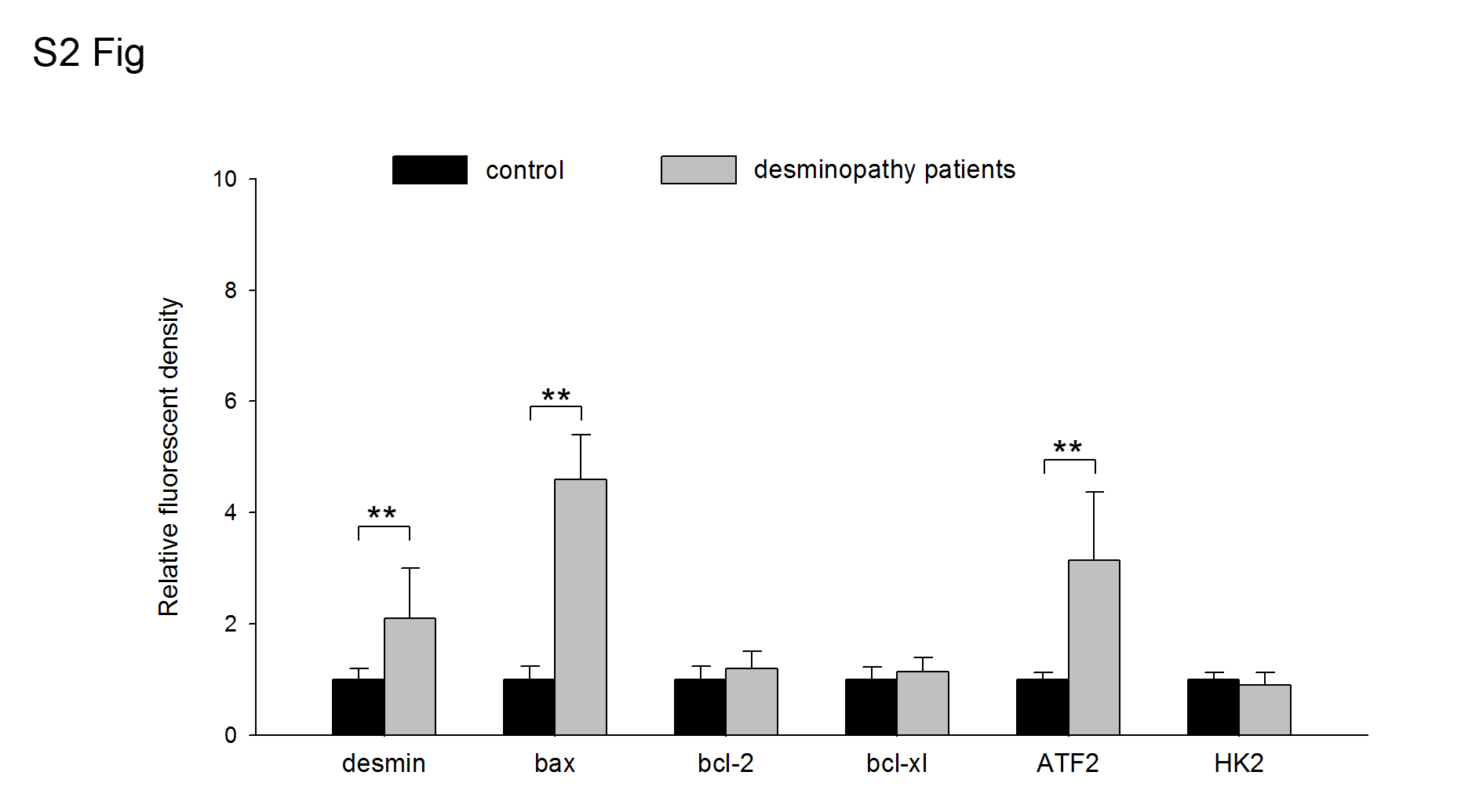

Supplement: S2 Fig — Statistical analysis of Fig 2. (TIF) [file pone.0167908.s002.tif]

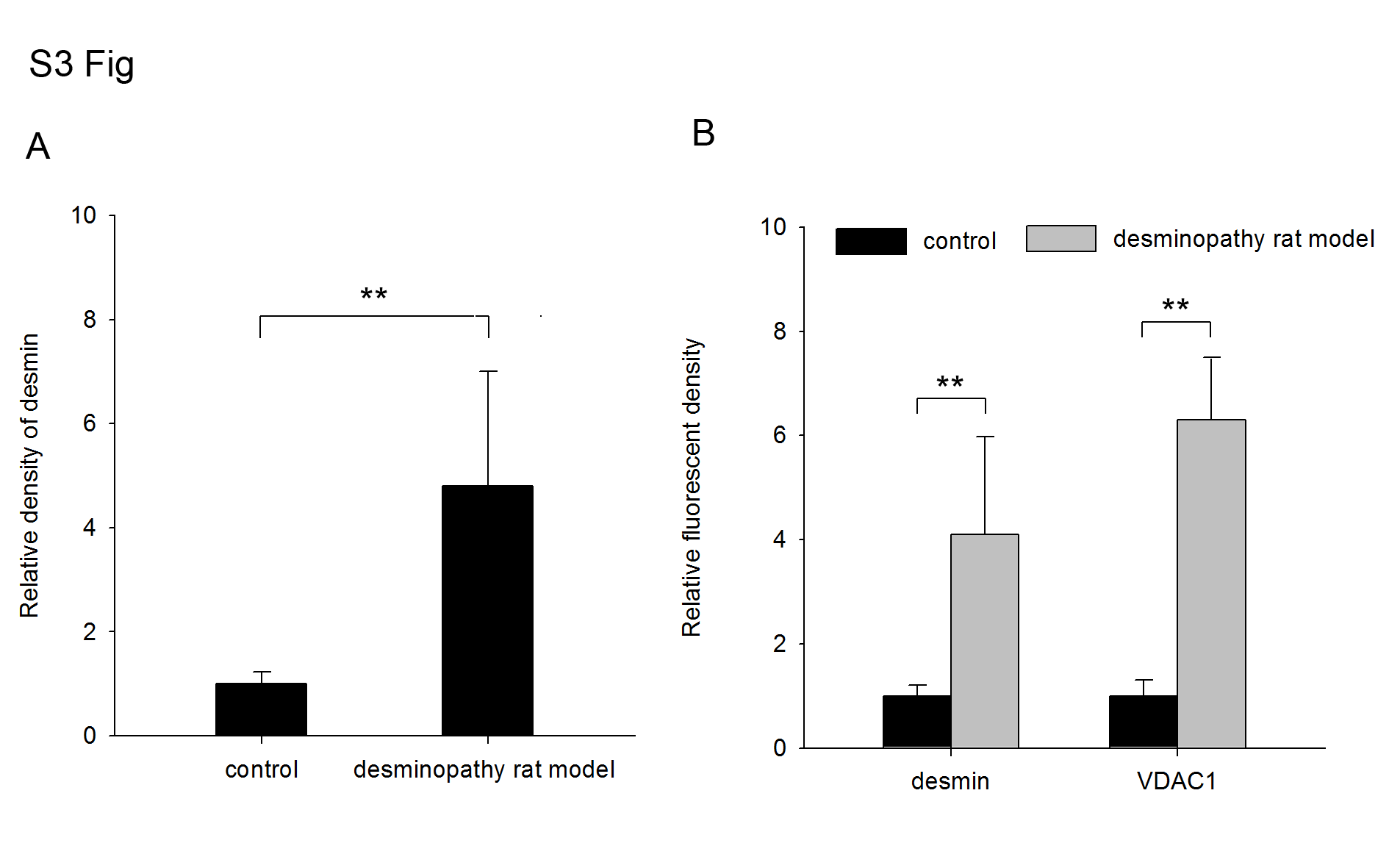

Supplement: S3 Fig — (A)Statistical analysis of Fig 3A. (B)Statistical analysis of Fig 3C. (TIF) [file pone.0167908.s003.tif]

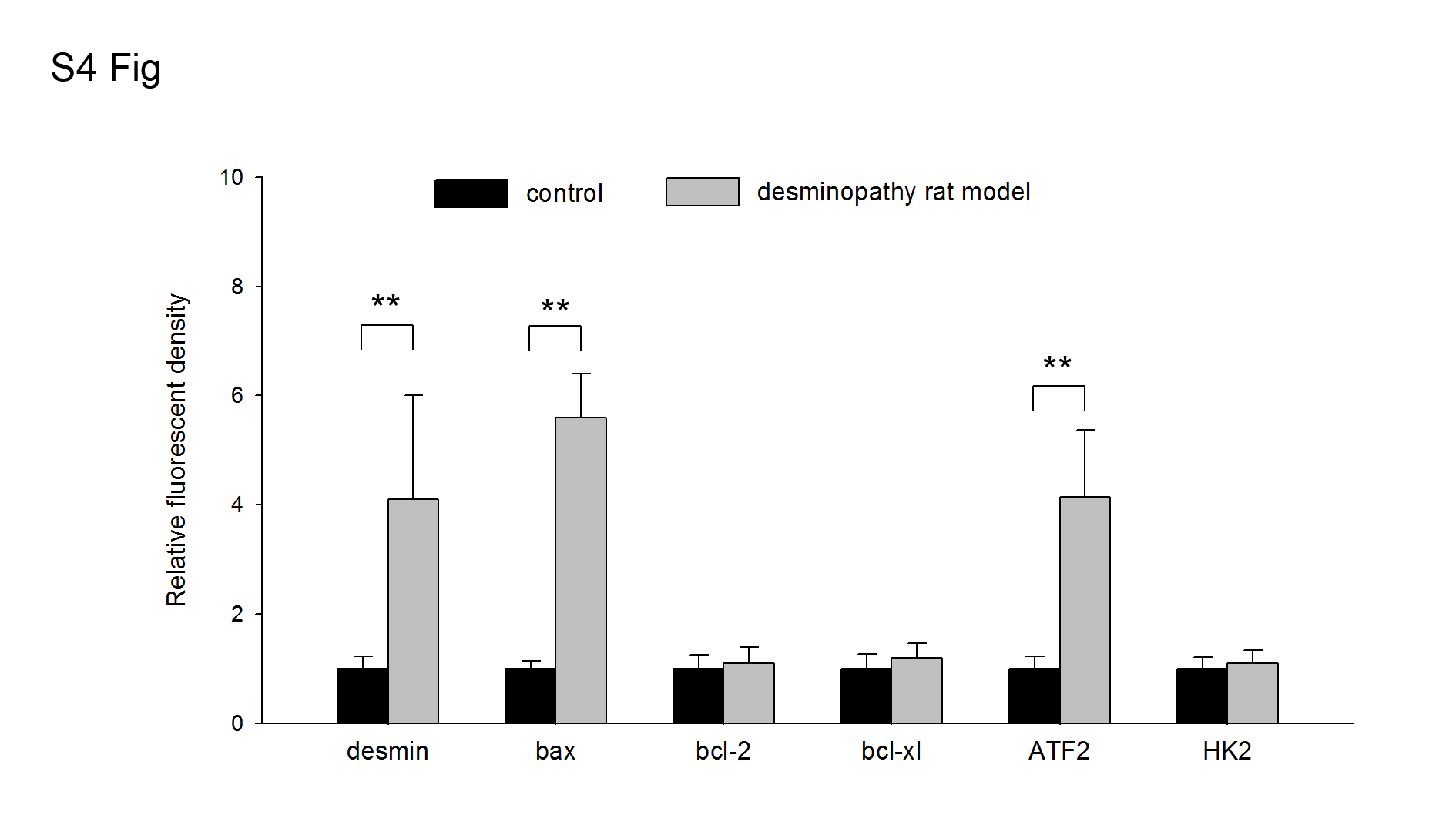

Supplement: S4 Fig — Statistical analysis of Fig 4. (TIF) [file pone.0167908.s004.tif]

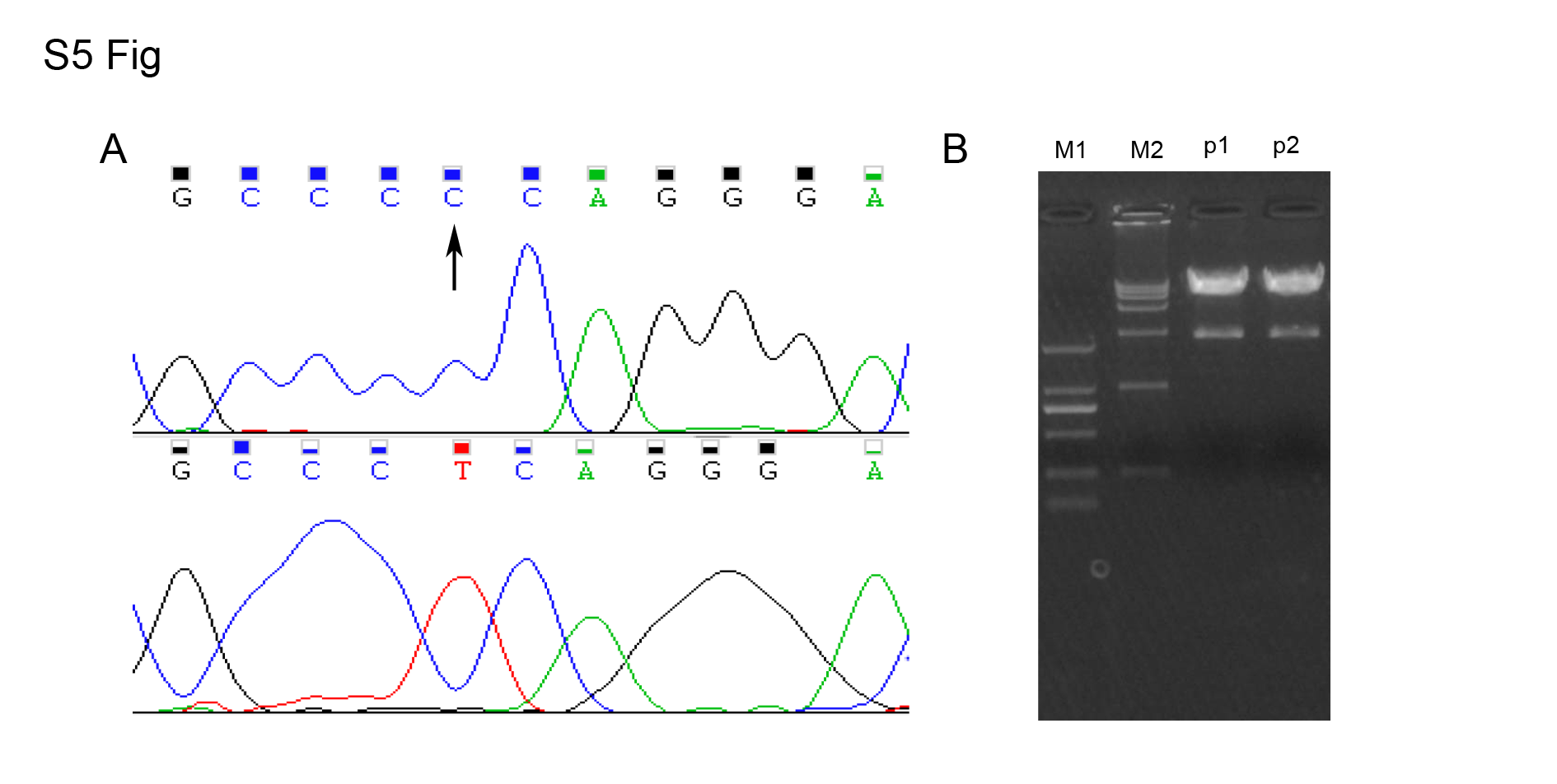

Supplement: S5 Fig — The point mutation of desmin as shown at the arrow (A),mutation position: c.821T>C, L2Ad5-DES as shown in (B), M1: Wide Range 2000 Marker(Takara):2kb,1kb,750bp,500bp,250bp,100bp; M2: Wide Range 500–15000 Marker(Takara): 15kb, 8kb, 5kb, 2.5kb, 1kb, 0.5kb; p1: the identification of rAd5-DES with restrictive endonuclease XbaI; p2: repeated p1. (TIF) [file pone.0167908.s005.tif]

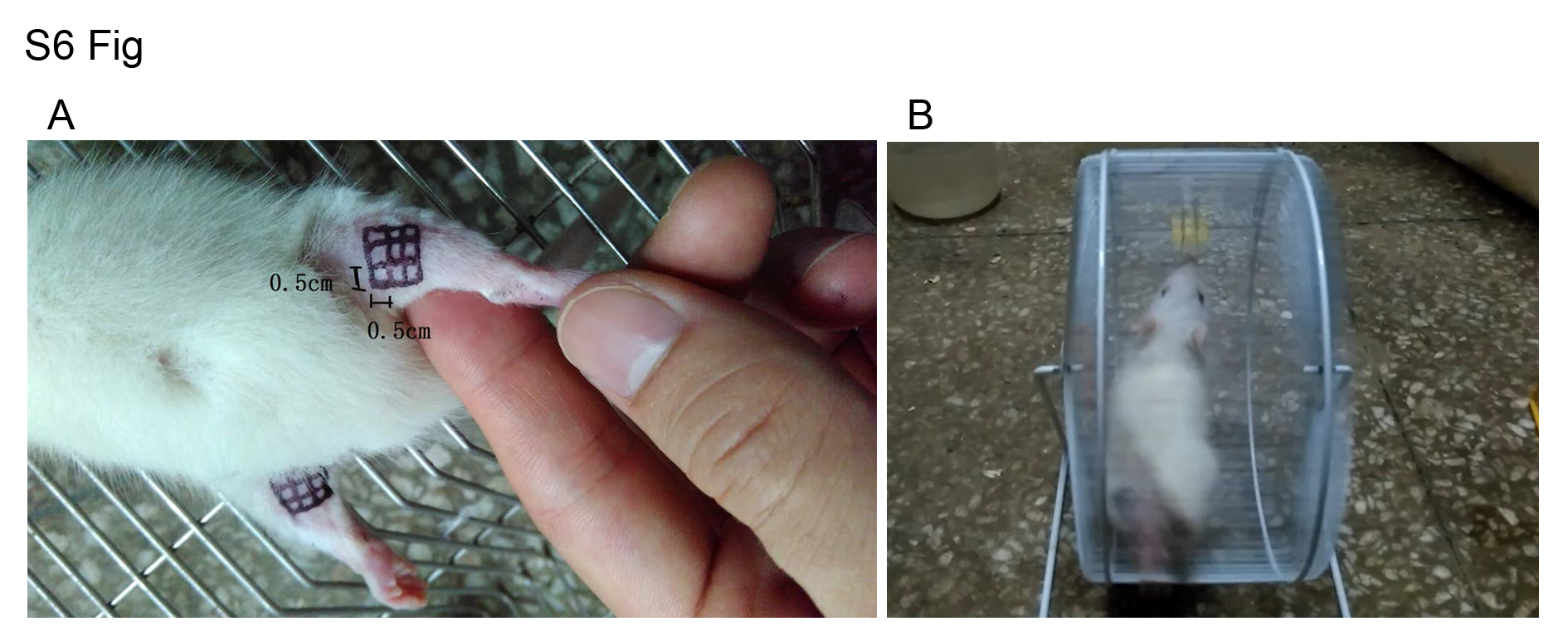

Supplement: S6 Fig — The 9 grids’ area of intramuscular injection was shown at (A), then the rats run at the designed time in the wheel everyday (B). (TIF) [file pone.0167908.s006.tif]
