# Supplementary material for: Voltage-Dependent Anion Channel 1(VDAC1) Participates the Apoptosis of the Mitochondrial Dysfunction in Desminopathy
Source: PLoS One. 2016 Dec 12;11(12):e0167908. doi: 10.1371/journal.pone.0167908 (PMC5152834; doi:10.1371/journal.pone.0167908)
Supplement: S1 Table — (DOCX) [file pone.0167908.s007.docx]

**S1 Table.** The information of desminopathy patients in our hospital.

| **Patient** | **Age(years)** | **Age at onset(years)** | **Gender** | **Preliminary diagnosis** | **Family History** |
| --- | --- | --- | --- | --- | --- |
| 1 | 21 | 20 | Female | Muscle weakness | No |
| 2 | 38 | 19 | Male | Muscular atrophy | No |
| 3 | 35 | 30 | Female | Muscle weakness | No |
| 4 | 41 | 37 | Female | Myotonic Muscular Dystrophy | Yes |
| 5 | 65 | 55 | Male | Muscle weakness | No |
| 6 | 23 | 13 | Female | Muscular dystrophy | No |
| 7 | 39 | 29 | Male | Facioscapulohumeral Muscular Dystrophy | No |
| 8 | 59 | 49 | Male | Myotonic Muscular Dystrophy | No |
| 9 | 30 | 25 | Male | Becker Muscular dystrophy | No |
| 10 | 49 | 44 | Male | Muscular atrophy | No |
| 11 | 59 | 47 | Female | Muscular dystrophy | No |
| 12 | 62 | 58 | Female | Muscular atrophy | No |
| 13 | 38 | 38 | Female | Muscle weakness | Yes |
| 14 | 29 | 28 | Male | Muscular atrophy | No |
